# Globally Important Haptophyte Algae Use Exogenous Pyrimidine Compounds More Efficiently than Thiamin

**DOI:** 10.1128/mBio.01459-17

**Published:** 2017-10-10

**Authors:** Magdalena A. Gutowska, Brateen Shome, Sebastian Sudek, Darcy L. McRose, Maria Hamilton, Stephen J. Giovannoni, Tadhg P. Begley, Alexandra Z. Worden

**Affiliations:** a Monterey Bay Aquarium Research Institute (MBARI), Moss Landing, California, USA; b Department of Chemistry, Texas A&M University, College Station, Texas, USA; c Department of Microbiology, Oregon State University, Corvallis, Oregon, USA; d Integrated Microbial Biodiversity Program, Canadian Institute for Advanced Research, Toronto, Ontario, Canada; University of California, Irvine

**Keywords:** algal evolution, haptophytes, pathway gaps, phytoplankton ecology, thiamin, vitamin biosynthesis, vitamin exchanges

## Abstract

Vitamin B_1_ (thiamin) is a cofactor for critical enzymatic processes and is scarce in surface oceans. Several eukaryotic marine algal species thought to rely on exogenous thiamin are now known to grow equally well on the precursor 4-amino-5-hydroxymethyl-2-methylpyrimidine (HMP), including the haptophyte *Emiliania huxleyi*. Because the thiamin biosynthetic capacities of the diverse and ecologically important haptophyte lineage are otherwise unknown, we investigated the pathway in transcriptomes and two genomes from 30 species representing six taxonomic orders. HMP synthase is missing in data from all studied taxa, but the pathway is otherwise complete, with some enzymatic variations. Experiments on axenic species from three orders demonstrated that equivalent growth rates were supported by 1 µM HMP or thiamin amendment. Cellular thiamin quotas were quantified in the oceanic phytoplankter *E. huxleyi* using the thiochrome assay. *E. huxleyi* exhibited luxury storage in standard algal medium [(1.16 ± 0.18) × 10^−6^ pmol thiamin cell^−1^], whereas quotas in cultures grown under more environmentally relevant thiamin and HMP supplies [(2.22 ± 0.07) × 10^−7^ or (1.58 ± 0.14) × 10^−7^ pmol thiamin cell^−1^, respectively] were significantly lower than luxury values and prior estimates. HMP and its salvage-related analog 4-amino-5-aminomethyl-2-methylpyrimidine (AmMP) supported higher growth than thiamin under environmentally relevant supply levels. These compounds also sustained growth of the stramenopile alga *Pelagomonas calceolata*. Together with identification of a salvage protein subfamily (TENA_E) in multiple phytoplankton, the results indicate that salvaged AmMP and exogenously acquired HMP are used by several groups for thiamin production. Our studies highlight the potential importance of thiamin pathway intermediates and their analogs in shaping phytoplankton community structure.

## IMPORTANCE

The concept that vitamin B_1_ (thiamin) availability in seawater controls the productivity and structure of eukaryotic phytoplankton communities has been discussed for half a century. We examined B_1_ biosynthesis and salvage pathways in diverse phytoplankton species. These comparative genomic analyses as well as experiments show that phytoplankton thought to require exogenous B_1_ not only utilize intermediate compounds to meet this need but also exhibit stronger growth on these compounds than on thiamin. Furthermore, oceanic phytoplankton have lower cellular thiamin quotas than previously reported, and salvage of intermediate compounds is likely a key mechanism for meeting B_1_ requirements under environmentally relevant scenarios. Thus, several lines of evidence now suggest that availability of specific precursor molecules could be more important in structuring phytoplankton communities than the vitamin itself. This understanding of preferential compound utilization and thiamin quotas will improve biogeochemical model parameterization and highlights interaction networks among ocean microbes.

## INTRODUCTION

Thiamin (vitamin B_1_) is an essential cofactor for multiple enzymes in central metabolism (1). In marine ecosystems, the possibility that thiamin is an important regulator of microbial communities, particularly phytoplankton, has been considered for decades. Initial estimates of thiamin concentrations in the oceans suggested they were extremely low. This finding has now been confirmed with more precise measurements that show thiamin is often at or below modern detection limits (approximately ≤0.81 pmol liter^−1^) and has complex distribution patterns in the photic zone (2–4). Comparative genome analyses between model taxa and marine algae have improved characterization of the thiamin biosynthesis pathway in important plankton groups (2, 5–7). These investigations demonstrated that complete, canonical biosynthesis pathways are missing in multiple eukaryotic phytoplankton groups, suggesting that thiamin auxotrophy is common and that thiamin may play a role in the regulation of primary production and phytoplankton community structure (5, 8–10). However, recent pathway gap analyses and experiments have also highlighted complexity in thiamin metabolism strategies among marine taxa (2, 6, 7, 11, 12).

Three widespread marine eukaryotic phytoplankton species have now been shown to obviate their requirement for exogenous thiamin by taking up the intermediate precursor compound 4-amino-5-hydroxymethyl-2-methylpyrimidine (HMP) (6, 11). Additionally, some algae may take up an intermediate compound to fulfill the thiazole side of the pathway (7). Previously, thiamin auxotropy had been reported based on amendment experiments using thiamin alone, although early studies indicated that 4-amino-5-aminomethyl-2-methylpyrimidin (AmMP), an analog of HMP, could be used by some “thiamin auxotrophic” algae (13, 14). Differences in how thiamin requirements are met extend beyond marine algae to the abundant oligotrophic bacterium SAR11, which requires HMP for growth and cannot use exogenous thiamin (2). To date, few measurements of thiamin precursors and their analogs have been made in the field. A pair of profiles from the oligotrophic Sargasso Sea show relatively consistent thiamin concentrations at dawn and dusk across photic zone depths but lower HMP concentrations at the surface than depth, possibly indicating higher biological utilization of HMP than of thiamin (2). At sites in the eastern Atlantic Ocean, offshore from Morocco, thiamin concentrations (33 to 457 pM) were higher than in the Sargasso Sea, while HMP concentrations were more similar (2.5 to 28 pM) to Sargasso Sea concentrations, and both compounds were lower at the chlorophyll maximum than at the surface (4). Collectively, these studies point to complex diversification in strategies for thiamin cycling and thiamin-related compound exchanges between marine microbial taxa.

Eukaryotic phytoplankton in the ocean are extremely diverse, and different lineages acquired photosynthesis through various endosymbiosis events (12). Thus, the genomes from the original host and the endosymbiosed organisms are “combined” in a single cell, introducing complexity to some biochemical pathways. Complete genome sequences are available for approximately 20 eukaryotic marine phytoplankton, many of which are prasinophytes with reduced genomes from the class *Mamiellophyceae* (also known as class II prasinophytes), or are from diatoms. The latter are thiamin prototrophs, while the *Mamiellophyceae* lack the complete set of enzymes known from canonical thiamin biosynthesis pathways (6). Haptophytes are another important group of marine primary producers (15–17) for which there are just two genome sequences available: one from *Emiliania huxleyi* (18) and the other from a freshwater harmful algal bloom species, *Chrysochromulina tobin* (19). The diversity of haptophytes as a whole (15, 20) makes extrapolation of thiamin pathways from analysis of the one genome-sequenced species examined to date, *E. huxleyi* (6), tentative at best.

Canonical thiamin biosynthesis pathways have two major precursors (HMP and 4-methyl-5-β-hydroxyethylthiazole [HET]) that are independently synthesized and, following phosphorylation, condensed to generate thiamin monophosphate (TMP) (1). Subsequent dephosphorylation and phosphorylation steps generate the biologically active cofactor thiamin pyrophosphate (TPP). A related pathway involves extra- and/or intracellular remodeling of pyrimidine compounds to generate HMP. Generation of pyrimidine compounds that enter the remodeling pathway has only been described for thiamin salvage processes in the bacterium *Bacillus subtilis* (21). These involve the destruction of the sulfur-containing heterocyclic ring of thiamin and subsequent generation of the pyrimidine analog *N*-formyl-4-amino-5-(aminomethyl)-2-methylpyrimidine (FAMP) (21, 22). Salvage of the hydrolysis product requires the removal of an amido group to generate AmMP and subsequent removal of an amino group to generate HMP. The amidohydrolase YLMB is responsible for the remodeling of FAMP in bacteria. In 2014, Zallot et al. demonstrated that a TENA protein subfamily (TENA_E) acts as an amidohydrolase in plants, fulfilling the same biochemical function as YLMB (23). In light of these new findings, a comparative analysis of TENA proteins in phytoplankton can be used to provide insight into the biochemical repertoire of thiamin salvage via pyrimidine remodeling. The distribution of genes encoding proteins involved in thiamin salvage processes has yet to be examined in eukaryotic phytoplankton, and use of pyrimidine analogs has not been rigorously tested across diverse taxa.

In addition to the above knowledge gaps on thiamin biosynthesis and salvage, actual cellular quotas of thiamin are unknown for oceanic phytoplankton. Cellular thiamin quotas have only been quantified in prototrophic phytoplankton isolates from brackish waters (24), and quotas for pelagic species come from estimates of minimum cellular quotas calculated from dose-response plots (11, 25). The efficacy of the latter approach has been disputed (26), leading to the development of direct chemical assays for seawater (2, 4) and/or use of other direct methods to measure thiamin in algal cells (24, 27). Moreover, thiamin quotas at growth rates where cell division still occurs (division quotas, as opposed to near-stationary-phase quotas), have rarely been measured and are not yet available for environmentally relevant taxa.

In this study, we identified and compared thiamin biosynthesis pathways in available haptophyte transcriptomes (28) and genomes (18, 19). Although the thiamin biosynthesis pathways are otherwise complete, the six orders studied appear to lack HMP synthase and show complexity in enzyme usage in the thiazole side of the pathway. We also analyzed the pyrimidine remodeling pathway and found the *TENA_E* gene subfamily in all six haptophyte orders studied. Laboratory experiments using cultures from two haptophyte classes (*E. huxleyi*, *Pavlova* sp., and *Prymnesium parvum*) demonstrated that at environmentally relevant concentrations, HMP as well as AmMP yields higher growth rates than thiamin. These haptophytes exhibited growth rates similar to those of the stramenopile *Pelagomonas calceolata* and several prasinophyte algae when grown on thiamin supplies in standard medium. We also quantified cellular thiamin quotas in a pelagic eukaryotic phytoplankton species for the first time using cultures grown under a range of thiamin and HMP availabilities. Our studies highlight complexity in thiamin cycling strategies and suggest that intermediate pyrimidine compounds may serve as a metabolic currency that shapes community interconnectivity.

## RESULTS

### Thiamin biosynthesis and salvage pathways in the context of haptophyte diversity.

**FIG 1:**
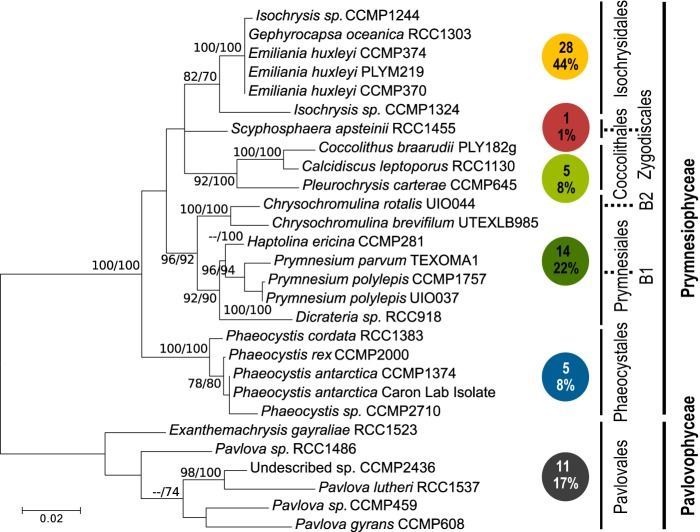
Relationships between the cultured haptophyte lineages analyzed here. This 18S rRNA tree was constructed using maximum likelihood methods and an alignment of nearly full-length gene sequences. The 28 species analyzed are distributed (numbers in colored circles) through the six orders. Two undescribed strains, CCMP2000 and CCMP2436, grouped in supported clades within the *Phaeocystales* and *Pavlovales* orders, respectively. Percentages of the MMETSP haptophyte transcriptomes are indicated for each order (bottom number in colored circles). Note that 12 species had ≥3 sequenced transcriptomes. Bootstrap support (100 replicates) is shown in the order maximum likelihood/neighbor joining for values of ≥70%. Note the *Syracosphaerales* order (not shown) is not represented in the MMETSP database and currently has no genome-level information available.

The six haptophyte orders studied here were represented by 28 species (58 transcriptomes in total) that covered the majority of haptophyte diversity present in culture. A phylogenetic reconstruction based on the 18S rRNA gene delineated the six orders (Fig. 1). The tree topology was consistent with current species assignments in haptophyte taxonomy wherein the *Pavlovophyceae* class is sister to the *Prymnesiophyceae* class, with the latter containing the *Phaeocystales*, *Prymnesiales*, *Coccolithales*, *Zygodiscales*, and *Isochrysidales* (29) (Fig. 1).

**FIG 2:**
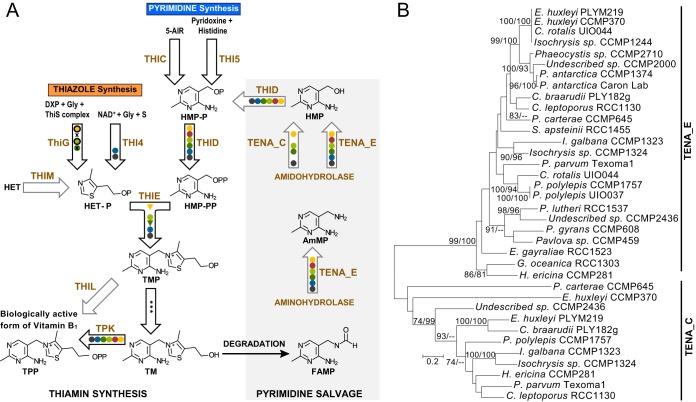
(A) Haptophyte thiamin biosynthesis and salvage enzymes. Enzymes (brown) and compounds (black) in the biosynthesis pathway based on reference 1 are shown with enzyme presence (solid circles) indicated for representatives of six haptophyte orders (colors as in Fig. 1); black outlines on circles indicate the gene is in the chloroplast genome, and triangles represent the presence of the THID and THIE fusion protein (TH1). Gray arrows show nonessential or salvage-related pathway steps (also with gray background, right side of panel). Haptophyte orders for which chloroplast genomes are not available are indicated by “x.” Unspecific phosphatases are represented by *. 5-AIR, 5-aminoimidazole ribotide; DXP, 1-deoxy-d-xylulose 5-phosphate; Gly, glycine; NAD^+^, NAD; S, reduced sulfur species; TM, thiamin; P, phosphate group; PP, pyrophosphate; HET, 4-methyl-5-β-hydroxyethylthiazole; HMP, 4-amino-5-hydroxymethyl-2-methylpyrimidine; AmMP, 4-amino-5-aminomethyl-2-methylpyrimidine; FAMP, *N*-formyl-4-amino-5-(aminomethyl)-2-methylpyrimidine. (B) Unrooted maximum likelihood phylogeny of haptophyte TENA_E and TENA_C proteins based on 390 amino acid positions. Bootstrap support (100 replicates) is shown in the order maximum likelihood/neighbor joining for values of ≥70%.

The thiamin biosynthesis pathway was nearly complete in all six haptophyte orders (Fig. 2A). The pyrimidine precursor synthase (THIC, as well as THI5) was the only key enzyme consistently absent from the predicted Marine Microbial Eukaryote Transcriptome Sequencing Project (MMETSP) protein sets and transcript assemblies. This enzyme was also absent from the genome of the *Prymnesiales* clade B2 species *C. tobin* (Fig. 2A; see Tables S1c and S1d in the supplemental material), similar to results from the *E. huxleyi* genome (6). Enzymes involved in further phosphorylation of HMP (THID) and condensation of HMP-PP with HET-P (THIE) to form thiamin monophosphate were identified in all six orders (Fig. 2). THID and THIE sequences occurred as the fusion protein TH1 (30) in *Isochrysidales*, *Coccolithales*, and *Prymnesiales* (clade B2) but were not fused in the *Pavlovales*, *Phaeocystales*, and *Prymnesiales* (clade B1). Synthesis of the thiazole precursor was encoded by the HET-P synthase THI4 (31) in the *Pavlovales* and *Phaeocystales*, whereas the *Prymnesiales* (B2 clade) and *Isochrysidales* orders contained the HET-P synthase thiG encoded on the chloroplast genome (Fig. 2; Table S1c). Chloroplast genomes are not presently available for the *Prymnesiales* (B1 clade) and *Coccolithales* orders. A short fragment of a HET kinase (putative THIM) was found only in one *Phaeocystales* species, but not other haptophytes (Table S1c). Furthermore, thiamin monophosphokinase (THIL) proteins were not found in the predicted peptides or transcriptome assemblies. Thiamin pyrophosphokinase (TPK) was present in representatives from all six haptophyte orders.

10.1128/mBio.01459-17.8 TABLE S1  Summary of thiamin-related genes in MMETSP haptophyte transcriptomes and a selection of genomes of sequenced organisms, including information used in Fig. 5, as well as measured and calculated thiamin cellular quotas in haptophytes. Download TABLE S1, XLSX file, 5 MB. Copyright © 2017 Gutowska et al. 2017 Gutowska et al. This content is distributed under the terms of the Creative Commons Attribution 4.0 International license.

We also investigated thiamin salvage proteins in the haptophytes. TENA_E was present in *E. huxleyi* and *C. tobin* as well as predicted MMETSP protein sets from all six orders (Fig. 2). TENA_C was present only in the *Isochrysidales*, *Coccolithales*, *Prymnesiales* (clade B1), and *Pavlovales* orders. Sequence conservation in the active and binding sites of TENA_C and TENA_E transcripts were congruent with established eukaryotic models (23, 32). The active-site cysteine residue identified in the bacterium *B. subtilis* TENA (Cys^135^) (22) was conserved in haptophyte TENA_C proteins. Haptophyte TENA_E sequences were similar to those of plants and cyanobacteria (23), which have only one of the double glutamate residues identified in the active site of TENA from the archaeon *Pyrococcus furiosus* (Glu^128–129^) (33). The glutamate residue corresponding to Glu^129^ in *P. furiosus* (Glu^140^ in *A. thaliana*) was consistently conserved in haptophyte TENA_E proteins. Phylogenetic analyses showed that TENA_E and TENA_C proteins clustered in two distinct well-supported groups (Fig. 2B). Clustering at the terminal nodes within the TENA_E clade corresponded to taxonomic relationships, but support was lacking at several more basal nodes (Fig. 2B). The broad distribution of TENA_E proteins observed here indicates that the amido- and aminohydrolase activities necessary for the hydrolysis of FAMP and AmMP are present across the six haptophyte orders studied (Fig. 2).

### Thiamin and pyrimidine precursor amendments in standard culture medium.

**FIG 3:**
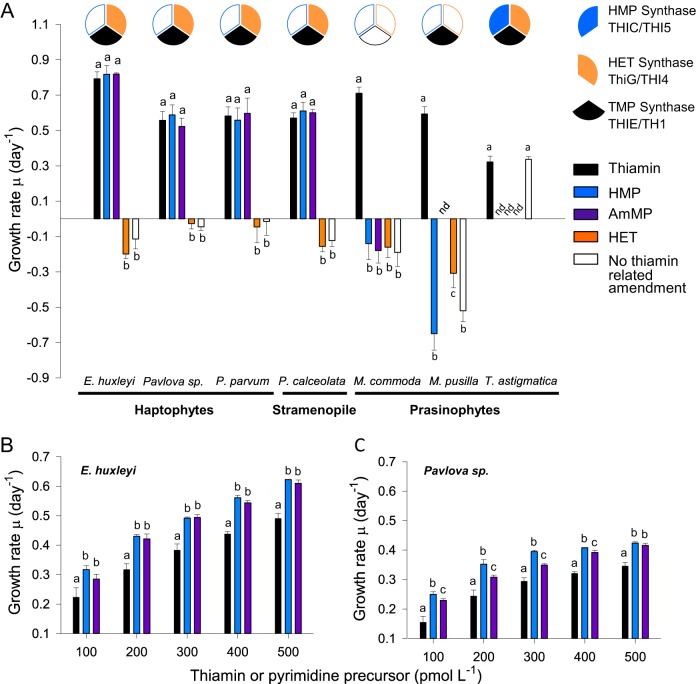
Phytoplankton growth rates on thiamin or pyrimidine compounds. (A) Acclimated mid-exponential growth rates of three haptophyte species, as well as the stramenopile *P. calceolata* and prasinophytes *M. commoda* and *M. pusilla* in response to 1 µM substrate additions. The negative control and the HET-amended treatment did not support growth. Pie charts illustrate the presence or absence of the three primary synthase enzymes in the thiamin biosynthesis pathway. (B and C) Exponential-phase growth rates of (B) *E. huxleyi* and (C) *Pavlova* sp. in grown-out cultures amended with 100 to 500 pM thiamin, HMP, or AmMP. Controls and treatments were performed in biological triplicates, and error bars represent the standard deviation (SD). Treatments that are not significantly different from one another share the same letter. nd, not determined.

Commonalities in haptophyte thiamin biosynthesis and pyrimidine remodeling pathways suggested the six orders have overarching similarities in thiamin and precursor utilization. Experiments with axenic *E. huxleyi* (*Isochrysidales*), *Pavlova* sp. strain CCMP459 (*Pavlovales*), and *P. parvum* (*Prymnesiales*) cultures demonstrated that these species grow under thiamin-depleted conditions when the precursor compound HMP or AmMP is present (Fig. 3A). As expected, the thiazole precursor (HET) did not support growth, nor did the negative control (no amendment). *E. huxleyi* growth rates with 1 µM HMP (0.82 ± 0.05 day^−1^), AmMP (0.82 ± 0.01 day^−1^), and thiamin (0.80 ± 0.03 day^−1^) amendments were not significantly different (*P* > 0.05). Likewise, *Pavlova* sp. strain CCMP459 growth rates in 1 µM thiamin amendment (0.56 ± 0.05 day^−1^) did not significantly differ from HMP (0.59 ± 0.06 day^−1^)- and AmMP (0.52 ± 0.05 day^−1^)-amended treatments. The comparable growth of *Pavlova* sp. strain CCMP459 in thiamin- and HMP-amended media complements prior results for *Pavlova lutheri* (11). *P. parvum* growth rates in the 1 µM thiamin amendment (0.58 ± 0.05 day^−1^) also were not significantly different from those in the HMP (0.56 ± 0.07 day^−1^)- and AmMP (0.60 ± 0.09 day^−1^)-amended treatments. Thus, the taxa tested here can utilize thiamin or pyrimidine precursor compounds to independently fulfill vitamin B_1_ cellular requirements.

To place our haptophyte results in a broader context, we also performed experiments with the stramenopile *P. calceolata* as well as prasinophytes that have no pathway gaps (*Tetraselmis astigmatica*) or have multiple different pathway gaps (*Micromonas commoda* and *Micromonas pusilla*). *P. calceolata* lacks THIC and grew well in media amended with either thiamin (0.57 ± 0.03 day^−1^) or HMP (0.61 ± 0.05 day^−1^), similar to the results from reference 11. Additionally, we found that amendment with AmMP supports comparable growth rates (0.60 ± 0.02 day^−1^), similar to our haptophyte results (Fig. 3A). *T. astigmatica* grew equally well with or without thiamin amendment (Fig. 3A). Neither *Micromonas* species grew in medium with HMP, AmMP, or HET, but both grew when thiamin was provided (Fig. 3A). These species differ with respect to the thiamin pathway. *M. pusilla* contains TMP synthase and two TPP-regulated transporters of unknown function, while *M. commoda* lacks these genes: both lack THIC and several other pathway elements (see Fig. S1 in the supplemental material). A second experiment tested growth on thiamin, HET, and/or HMP or no thiamin-related amendment in L1 medium with Sargasso Sea water as the base instead of artificial seawater (ASW) (see Text S1 in the supplemental material for methods). *M. commoda* grew best in thiamin amended medium, while *M. pusilla* grew as well or better without added thiamin (see Fig. S2 and Fig. S3 in the supplemental material). After 92 h, *M. pusilla* treatments without thiamin had higher average cell yields than thiamin-amended treatments at 1 and 0.3 µM. Growth and cell yields for the HET- and HMP-amended treatments were intermediate to the thiamin and unamended treatments (Fig. S2). Precursor-related compounds present in the seawater may therefore have obviated the need in *M. pusilla* for the precursor synthases, similar to findings for a related prasinophyte, *Ostreococcus* (7).

10.1128/mBio.01459-17.1 TEXT S1  Supplemental methods. Download TEXT S1, DOCX file, 0.02 MB. Copyright © 2017 Gutowska et al. 2017 Gutowska et al. This content is distributed under the terms of the Creative Commons Attribution 4.0 International license.

10.1128/mBio.01459-17.2 FIG S1  Green algal lineages analyzed here in MMETSP data. The 18S rRNA tree was constructed using maximum likelihood methods and an alignment of nearly full-length gene sequences. The strains analyzed are distributed between the seven lineages of prasinophytes, which diverge roughly at the class level and chlorophytes. Thiamin biosynthesis and salvage enzymes are denoted with shapes described on the figure (Table S2). Results from genomic databases are denoted in yellow, and transcriptomes from the MMETSP database are highlighted in light blue. Classes are *sensu* Duanmu, Bachy et al. 2014 (58). Download FIG S1, PDF file, 0.1 MB. Copyright © 2017 Gutowska et al. 2017 Gutowska et al. This content is distributed under the terms of the Creative Commons Attribution 4.0 International license.

10.1128/mBio.01459-17.3 FIG S2  Abundance of *M. pusilla* when supplied with thiamin or pyrimidine analogs in medium made with natural Sargasso Sea water. *M. pusilla* reached higher abundances in treatments without added thiamin and grew as well, or better, than those with added thiamin. The highest growth was observed in treatments without thiamin or precursor additions (0.51 ± 0.01 day^−1^, compared with 0.37 ± 0.01 day^−1^ for 1 µM thiamin treatments and 0.32 ± 0.02 day^−1^ for 0.3 µM thiamin treatments). Accordingly, after 92 h, treatments without thiamin also had higher average cell yields (final minus initial cell count). Yields were (1.97 × 10^7^) ± 454,707 cells ml^−1^ for no thiamin amendment compared to (9.06 × 10^6^) ± 668,780 cells ml^−1^ for 1 µM thiamin additions and (8.30 × 10^6^) ± 802,020 cells ml^−1^ for 0.3 µM thiamin additions. Controls and treatments were performed in biological triplicates, and error bars represent the SD of the triplicates. Download FIG S2, PDF file, 0.05 MB. Copyright © 2017 Gutowska et al. 2017 Gutowska et al. This content is distributed under the terms of the Creative Commons Attribution 4.0 International license.

10.1128/mBio.01459-17.4 FIG S3  Abundance of *M. commoda* when supplied with additional thiamin in medium made with natural Sargasso Sea water. Unlike the growth response observed in *M. pusilla*, *M. commoda* did not reach high densities in natural Sargasso Sea water over the 119-h time course unless thiamin was added. Treatments in this preliminary experiment were not replicated. Download FIG S3, PDF file, 0.04 MB. Copyright © 2017 Gutowska et al. 2017 Gutowska et al. This content is distributed under the terms of the Creative Commons Attribution 4.0 International license.

10.1128/mBio.01459-17.9 TABLE S2  Summary of thiamin-related genes or proteins they encode in MMETSP prasinophyte transcriptomes, including information used in Fig. 5, and query sequences used in BLAST searches. Download TABLE S2, XLSX file, 0.02 MB. Copyright © 2017 Gutowska et al. 2017 Gutowska et al. This content is distributed under the terms of the Creative Commons Attribution 4.0 International license.

### Thiamin and pyrimidine precursor use at environmentally relevant concentrations.

Growth rates under environmentally relevant concentrations of thiamin, HMP, and AmMP differed for the tested representatives of the *Prymnesiophyceae* and *Pavlovophyceae* classes (Fig. 3B and C). *E. huxleyi* growth rates increased linearly over amendments from 100 to 500 pM (Fig. 3B). At each concentration tested, growth rates of thiamin-amended cultures were significantly lower than those supplied with pyrimidine precursors (*P* < 0.001). Growth rates between HMP- and AmMP-amended cultures did not differ (*P* > 0.05). The *Pavlova* sp. strain CCMP459 growth rate showed a large step increase between the 100 and 200 pM amendments and only small increases at the higher concentrations. Additionally, while growth rates were still slowest with thiamin, they also differed between HMP and AmMP (*P* < 0.01). This indicates that these haptophytes take up or utilize the two pyrimidine precursors with different efficiencies at low concentrations. However, for both taxa, amendment with the pyrimidine precursors at environmentally relevant concentrations supported higher growth rates than thiamin (Fig. 3B and C).

Thiamin is reportedly degraded by sunlight (34) and more specifically, by UV radiation (35, 36). While the degradation products have not been characterized using modern methods, they have been proposed to be AmMP and other products based on colorimetric analyses (35). Therefore, in addition to comparing maximum cellular densities across treatments with 500 pM thiamin or HMP, we also used a thiamin-amended (500 pM) medium that had been exposed to UV light (Text S1). The maximum cellular density in the UV-exposed, thiamin-amended medium was significantly higher than in the undegraded thiamin treatment, but less than in the HMP treatment (*P* < 0.001) (see Fig. S4 in the supplemental material). These results suggest that the compound or compounds generated by UV degradation of thiamin were more readily transported or utilized by *E. huxleyi* than intact thiamin molecules.

10.1128/mBio.01459-17.5 FIG S4  Maximum cell densities of *E. huxleyi* in treatments amended with 500 pM thiamin or HMP. Additionally, in one of the treatments, 500 pM amended medium was irradiated for 30 min with UV light (312 nm) (Text S1). The maximum cell density reached in the HMP-amended treatment was approximately double that of the thiamin-amended treatment. Degradation of thiamin to pyrimidine compounds by UV exposure also led to a significantly greater maximum cell density compared to the nonirradiated thiamin treatment (*P* < 0.01). Controls and treatments were performed in biological triplicates, and error bars represent the SD of the triplicates. Download FIG S4, PDF file, 0.03 MB. Copyright © 2017 Gutowska et al. 2017 Gutowska et al. This content is distributed under the terms of the Creative Commons Attribution 4.0 International license.

### Calculated estimates versus measured MCQs.

**FIG 4:**
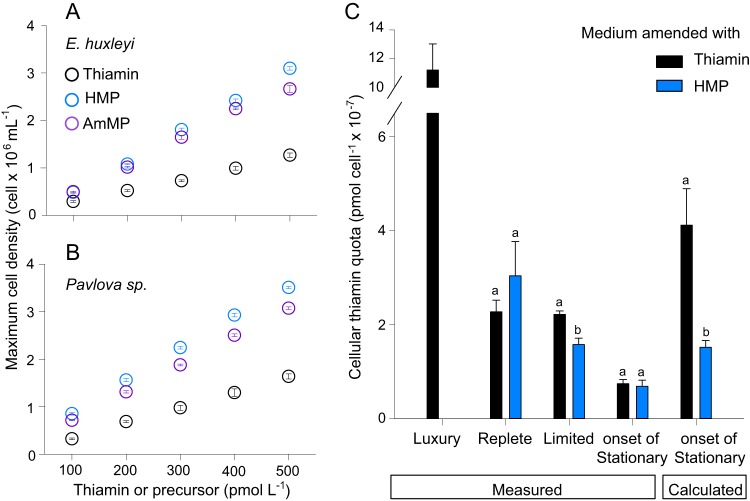
Haptophyte maximum cell densities and cellular thiamin quotas. Maximum cell densities were attained at the onset of the stationary phase in (A) *E. huxleyi* and (B) *Pavlova* sp. under amendments ranging from 100 to 500 pM thiamin (black circles), HMP (blue circles), and AmMP (purple circles). Controls and treatments were performed in biological triplicates, and error bars (within circles) represent the SD. Treatments that are not significantly different from one another share the same letter. Note that in ocean seawater, the maximum reported thiamin and HMP concentrations are 400 pM (4) and 35 pM (2), respectively. (C) Thiamin cellular quotas in *E. huxleyi* obtained by the thiochrome chemical assays of cultured cells grown in either thiamin (black bars) or HMP (blue bars) amended medium or by calculation (Table 1).

The minimum cellular quota (MCQ) of thiamin was estimated using established dose-response calculation methods (25) for the *Pavlovophyceae* and *Prymnesiophyceae* representatives. This quota represents the required intracellular thiamin pool for maintenance of biochemical and physiological processes but not for cell division. At the amendment concentrations used here, cell densities were limited by thiamin or precursor availability, as evidenced by the linear relationship between maximum cell density and the initial thiamin or precursor concentrations (Fig. 4A and B). The MCQ for cells grown with thiamin was greater than for those grown with HMP or AmMP, and varied significantly between species (*P* < 0.01). When grown on thiamin, the MCQs were estimated at (4.12 ± 0.78) × 10^−7^ pmol cell^−1^ (*E. huxleyi*, i.e., 248,000 molecules cell^−1^) and 3.08 ± 0.31 × 10^−7^ pmol cell^−1^ (*Pavlova* sp.) (Table S1g). MCQ estimates for cells grown on the pyrimidine precursors were significantly lower. In *E. huxleyi*, the MCQs were (1.52 ± 0.14) × 10^−7^ pmol cell^−1^ (HMP) and (1.79 ± 0.38) × 10^−7^ pmol cell^−1^ (AmMP). The calculated cellular thiamin quotas were also different between cells grown on HMP and AmMP, but not between species for an individual precursor: e.g., for HMP, the cellular thiamin quotas were (1.52 ± 0.14) × 10^−7^ pmol cell^−1^ (*E. huxleyi*) and (1.49 ± 0.18) × 10^−7^ pmol cell^−1^ (*Pavlova* sp.) (Table S1g).

We also used a chemical assay to quantify thiamin quotas in *E. huxleyi* cells that were harvested at the same growth phase as for the MCQ calculations (the onset of stationary phase). MCQs from direct measurements were not statistically different between cells grown in thiamin and HMP-amended medium (Table S1h). The MCQs were (6.1 ± 0.81) × 10^−8^ pmol cell^−1^ in the thiamin-amended treatment and (6.9 ± 0.31) × 10^−8^ pmol cell^−1^ in the HMP treatment. While these measurements were similar to each other, they were significantly lower than the estimated values (*P* < 0.001). Thus, thiamin MCQs can be substantially overestimated when they are calculated instead of measured (Table 1).

**TABLE 1:** Measured cellular quotas for TPP, TMP, and ThF in *E. huxleyi* CCMP2090^a^

Treatment	Initial concn (nmol liter^−1^)	Growth rate (day^−1^)	Mean quota per cell (pmol cell^−1^)				T sum/C (nmol g C^−1^)
			TPP	TMP	ThF	T sum	
Replete conditions							
Luxury thiamin	300	0.74 (0.05)	4.47 × 10^−7^ (3.66 × 10^−7^)	4.08 × 10^−7^ (3.62 × 10^−8^)	2.6 × 10^−7^ (1.48 × 10^−7^)	11.15 × 10^−7^ (1.83 × 10^−7^)	327.7 (62.5)
Replete thiamin	10	0.71 (0.04)	1.48 × 10^−7^ (4.00 × 10^−8^)	5.78 × 10^−8^ (3.49 × 10^−8^)	2.11 × 10^−8^ (1.05 × 10^−8^)	2.27 × 10^−7^ (2.48 × 10^−8^)	54.3 (4.7)
Replete HMP	10	0.73 (0.06)	1.43 × 10^−7^ (8.80 × 10^−8^)	1.42 × 10^−7^ (8.00 × 10^−8^)	1.94 × 10^−8^ (7.10 × 10^−9^)	3.04 × 10^−7^ (7.32 × 10^−8^)	79.5 (22.5)
Limiting conditions							
Limiting thiamin	0.5	0.25 (0.13)	1.60 × 10^−7^ (6.78 × 10^−9^)	2.89 × 10^−8^ (1.48 × 10^−8^)	3.31 × 10^−8^ (2.26 × 10^−8^)	2.22 × 10^−7^ (7.28 × 10^−9^)	31.6 (3.4)
Limiting HMP	0.1	0.21 (0.11)	1.14 × 10^−7^ (7.12 × 10^−9^)	2.08 × 10^−8^ (1.97 × 10^−9^)	2.30 × 10^−8^ (6.00 × 10^−9^)	1.58 × 10^−7^ (1.36 × 10^−8^)	28.9 (3.0)

a Numbers in parentheses represent the standard deviation from biological triplicates. C, carbon; T sum, the sum of the three thiamin states quantified (TPP, TMP, and ThF).

### Measured cellular thiamin quota for cells in exponential growth.

We measured thiamin quotas in cells grown in replete and limiting supplies of thiamin and HMP (Table 1; Fig. 4C). The luxury supply level was 300 nM thiamin, which is the standard concentration in most algal media. For the replete, but nonluxury mode, 10 nM thiamin was used (here referred to as replete). Growth rates were 0.74 ± 0.05 day^−1^ (luxury) and 0.71 ± 0.04 day^−1^ (replete), and the thiamin quota was higher in the former [(11.2 ± 1.83) × 10^−7^ pmol cell^−1^; 327.7 ± 110.6 nmol g C^−1^] than the latter [(2.27 ± 0.25) × 10^−7^ pmol cell^−1^; 54.3 ± 4.7 nmol g C^−1^]. The 5-fold difference in thiamin quotas between the two treatments demonstrated that luxury storage occurs under conditions of high thiamin availability (e.g., in standard culture medium). We also assayed cells grown in 10 nM HMP and found that their thiamin quota [(3.04 ± 0.730) × 10^−7^ pmol cell^−1^; 79.5 ± 22.5 nmol g C^−1^] was not significantly different (*P* > 0.05) from that of cells grown in the replete thiamin treatment.

To obtain quotas of greater environmental relevance, we quantified thiamin in cells grown under thiamin and HMP concentrations akin to those reported in the field (2–4). The average growth rate was 0.25 ± 0.13 day^−1^ over 10 generations in medium amended with 0.5 nM thiamin (Table 1; Fig. 4C). The thiamin cellular quota in this environmentally relevant “limited” treatment was 6-fold lower [(2.22 ± 0.07) × 10^−7^ pmol cell^−1^; 31.6 ± 3.4 nmol g C^−1^] than standard medium, and it was not statistically different from the per cell quotas assayed from cells grown in replete thiamin or HMP amendments. In the HMP-limited treatment (0.1 nM HMP), the growth rate averaged 0.21 ± 0.11 day^−1^, and the thiamin quota was lower [(1.58 ± 0.14) × 10^−7^ pmol cell^−1^; 28.9 ± 3.0 nmol g C^−1^]. Thus, the thiamin cellular quota for cells grown at environmentally relevant HMP concentrations was significantly lower than that of all other treatments (*P* < 0.001).

## DISCUSSION

Low environmental thiamin concentrations have long been hypothesized to exert a regulatory role over primary producers (5, 9, 10, 37). Recent research has highlighted the potential importance of direct HMP acquisition for use in thiamin synthesis when the synthase (THIC or THI5) is missing (2, 6). The few available measurements of HMP concentrations in the oligotrophic ocean demonstrate that HMP concentrations can exceed those of thiamin (2). Despite the fact that experiments involving thiamin amendment have been performed on at least 21 haptophyte species (6, 11, 25, 38, 39), use of the precursor compound HMP had only been examined in two (6, 11). Thus, the thiamin biosynthesis strategies of haptophytes still remain largely uncharacterized. This dearth of knowledge is problematic because haptophytes are widespread, can form a large fraction of phytoplankton biomass (15–17), and are diverse (20, 29). As seen in recent studies of differential use of B_12_ and analogs (40, 41), our research suggests that hidden specificities for thiamin-pathway-related compounds are involved in microbial interactions.

### Thiamin biosynthesis in the phylum *Haptophyta.*

Our transcriptome analysis illustrates high conservation in the thiamin biosynthesis pathway across the six haptophyte orders examined (Fig. 1). All orders appear to lack the known HMP synthases (THIC and THI5) but have otherwise complete biosynthesis pathways (Fig. 2). These findings are based on searches of predicted protein sets and coding sequences from the MMETSP transcriptomes as well as two sequenced genomes (Table S1a and b). While absence from transcriptome data does not ensure absence from the corresponding genome, the presence of other pathway components indicates the transcriptome assemblies had coverage of this pathway (Table S1c to f). It is unlikely that another enzyme is fulfilling a similar function, especially given our experimental results and the apparent absence of HMP synthase in the genomes of *E. huxleyi* (6) and *C. tobin* (Table S1). It is also unlikely that THIC-like sequences were not detected due to diversification. Synthesis of HMP-P from the 5-aminoimidazole ribotide (5-AIR) precursor by THIC is a complicated rearrangement reaction (1). Additionally, THIC exhibits notable amino acid conservation between the land plant *Arabidopsis thaliana* and bacteria such as *Escherichia coli* and *B. subtilis* (42). Indeed, we detected THIC in 12 major marine lineages, including diatoms, dinoflagellates, chlorophytes, and prasinophytes, using homology-based searches (Table S1i). The only other known mechanism for HMP synthesis, from pyridoxine and histidine in fungal lineages, has been attributed to THI5 (1), which we also did not detect. Our experiments with HMP, AmMP, and thiamin amendments on species from the *Isochrysidales*, *Prymnesiales*, and *Pavlovales* (Fig. 3A) further support the pathway analyses by demonstrating that while cells die with no amendment, availability of HMP (or AmMP) obviates the need for an external supply of the vitamin itself (Fig. 2A).

We observed variations in the arrangement of THID and THIE (encoded by independent genes or the fusion gene *Th1*) and variations in the enzymes for thiazole synthesis and phosphorylation (Fig. 2). With respect to thiazole, we found a plant-like thiazole synthase (THI4) in the two most ancient (43) haptophyte orders, the *Pavlovales* and *Phaeocystales*, and it is present in many other eukaryotes (6, 31). The other haptophyte orders contain an enzyme of bacterial (plastidial) origin, thiG, which is also present in diatoms and several other algal lineages (Table S1i). Additionally, based on our transcriptome and genome analyses, THIM appears to be absent from nearly all haptophytes. This finding should be interpreted with caution as many taxa are represented only by transcriptome data. However, it suggests that haptophytes do not salvage the unphosphorylated thiazole precursor, or if they do, it is accomplished by some as yet unknown protein. The presence of thiazole synthases along with the apparent absence of THIM suggests that the thiazole precursor is synthesized by haptophytes rather than salvaged from the environment.

### Pyrimidine utilization by diverse phytoplankton groups.

We extended our studies to include an important stramenopile alga and three prasinophyte species (Fig. 3A). Results from the stramenopile *P. calceolata* and those of a prior study (11) indicate that HMP is used by other eukaryotic phytoplankton as well. Moreover, *P. calceolata* can also remodel AmMP into the pyrimidine precursor to then synthesize thiamin (Fig. 3A). The prasinophytes tested showed varied strategies (Fig. 3A) and exhibited diverse presence/absence patterns for known thiamin biosynthesis pathway enzymes (see Fig. S1 and Table S2 in the supplemental material). Class II prasinophytes such as *Micromonas* have some of the most extensive pathway reductions among known marine phytoplankton (Fig. S1) (6). *M. commoda* does not possess HMP or HET synthases or thiamin monophosphate synthase (TH1) and is therefore entirely dependent on exogenous thiamin supplies (Fig. 3A). In contrast, *M. pusilla* appears to be auxotrophic for both precursor compounds, but does have TH1 as well as riboswitch-controlled transporters of unknown specificity. This led to the proposal that *M. pusilla* and *Ostreococcus*, which have the same pathway configuration, potentially utilize alternate, unknown compounds as thiamin precursor compounds (6). Neither *M. commoda* nor *M. pusilla* grew in culture studies where HMP and HET were cosupplied in artificial seawater (6). The *M. commoda* results here were the same in experiments in natural and artificial seawater, confirming reliance on external thiamin sources and that the low concentrations of thiamin in Sargasso seawater do not support growth (Fig. 3A; Fig. S2). In contrast, *M. pusilla* treatments without amendments supported the highest biomass, while those amended with HMP and HET or thiamin had lower growth rates and lower cell densities (Fig. S2). *M. pusilla* may therefore utilize low levels of environmental thiamin more efficiently than *M. commoda*, and reduced growth rates in amended medium would then have resulted from thiamin toxicity in *M. pusilla* but not *M. commoda*. Alternatively, one or more novel compounds may substitute for the standard precursors (or be remodeled to meet this need). Recent work on *Ostreococcus lucimarinus* indicates that an as yet unknown thiazole-related precursor, potentially synthesized by cocultured bacteria, is indeed used by *Ostreococcus* to meet thiamin requirements when HMP is also made available (7). This supports inferences from prior research suggesting THIM plays a role in salvage of exogenous thiazole compounds (6) and agrees with indications from our *M. pusilla* experiments (Fig. S2). In contrast, *M. commoda* does not appear to have the capacity to use exogenous intermediate compounds because it even lacks TMP synthase, as does the class II genus *Bathycoccus*, and exhibits differences in natural seawater experiments (Fig. S1 and S3). Together with results on the pyrimidine side of the pathway and use of degradation products in haptophytes, these findings indicate there is significant diversity in the thiamin precursor molecules and salvage in major marine lineages.

While the haptophytes and pelagophyte we tested used thiamin, HMP, and AmMP with similar efficiencies at standard medium amendment concentrations, this did not occur at more environmentally relevant concentrations (Fig. 3). Species from the *Prymnesiophyceae* (*E. huxleyi*) and *Pavlovophyceae* (*Pavlova* sp.) classes had higher mid-exponential growth rates in HMP- and AmMP-amended medium than on thiamin when supplied at lower concentrations (Fig. 3B and C). Additionally, for *E. huxleyi*, growth was equivalent with AmMP and HMP (Fig. 3B), whereas *Pavlova* sp. exhibited higher growth rates in HMP- than AmMP-amended media (Fig. 3C). For comparison, AmMP at environmentally relevant concentrations does not support growth of the bacterium SAR11 (2). Hence, it appears that among some marine bacteria, haptophytes, and potentially other eukaryotic phytoplankton, there are higher transport and/or utilization efficiencies for precursors than for thiamin. Specialization for different pyrimidine compounds and/or thiamin may therefore play an important role in niche partitioning and competition avoidance among plankton community members.

### Salvage of external pyrimidine compounds.

Phytoplankton species that rely on external HMP to fulfill thiamin requirements can acquire this precursor compound directly from seawater, but the possibility also exists that HMP is generated from other externally acquired pyrimidine compounds, which could then be remodeled by TENA proteins. We identified here for the first time that TENA proteins are not only present in all the haptophytes examined, but distinct TenA_C and TenA_E protein families occur (Fig. 2B). Based on the results of Zallot and colleagues (23), the latter should provide the necessary hydrolase for pyrimidine salvage in haptophytes. Furthermore, our experiments demonstrated that haptophytes grow in AmMP-amended medium, consistent with the interpretation that HMP can be generated by incorporation and remodeling of AmMP (Fig. 3A and C). An examination of TENA_E distributions in prokaryote genomes resulted in a proposed habitat bias connected to its occurrence and presumably the importance of pyrimidine salvage, with higher prevalence in taxa inhabiting environments where thiamin might be rapidly degraded (23). These patterns are suggestive of a TENA_E-based strategy in which thiamin degradation products are acquired and modified to meet thiamin requirements.

Sunlight, or possibly just the UV component, has the potential to degrade thiamin in the photic zone surface layer when the water column is highly stratified (34, 44). Our proof-of-concept experiment on growth of *E. huxleyi* on uncharacterized thiamin UV degradation products resulted in higher cell densities than in treatments where intact thiamin was provided (Fig. S4). This supports the conclusion that *E. huxleyi* may preferentially use precursor compounds over thiamin at picomolar concentrations. Salvage capabilities of marine phytoplankton groups may therefore provide additional strategies for meeting cellular thiamin requirements and could influence interactions in the surface ocean.

Upper ocean thiamin concentrations in the photic zone range from below detection (0.8 or 2.4 pM detection limit depending on the study) to ~400 pM in the few regions studied (2–4, 45). In the Sargasso Sea, HMP concentrations were higher than thiamin concentrations at several depths in the two profiles characterized (2), while in the eastern Atlantic, HMP concentrations were approximately an order of magnitude lower than thiamin concentrations at all photic zone depths examined (4). Thus, the greater efficiency in use of HMP (and AmMP) over thiamin observed here would confer an advantage in environments where standing stocks of thiamin were similarly low (e.g., <500 pM). Of course, standing stock measurements of vitamin concentrations in nature do not reflect flux, and high turnover rates have been reported in coastal waters (46). Thiamin, HMP, and AmMP concentrations and turnover rates, as well as those of other possible intermediate compounds, must be determined in more marine environments to gain a comprehensive view of compound exchanges within microbial communities.

### Quantification of thiamin cellular quotas.

Our first ever measurements of thiamin cellular quotas in an oceanic, eukaryotic phytoplankton species show that the quota is lower per cell than published estimates for haptophytes (11, 25) when the growth rate is limited by thiamin availability (Fig. 4C; Table 1). The cellular thiamin quotas are not significantly different between cells grown under limiting availability of thiamin versus HMP (Fig. 4C; Table 1). Cellular thiamin quotas are significantly higher when storage is possible, as demonstrated by the luxury treatment quota being 5-fold higher than the replete treatment quota in our experiments. Quotas have otherwise only been quantified in five brackish water eukaryotic phytoplankton species, all of which were grown under conditions of luxury thiamin availability (see Fig. S5 in the supplemental material). These were the diatoms *Phaeodactylum tricornutum* and *Skeletonema costatum*, the chlorophyte *Dunaliella tertiolecta*, the dinoflagellate *Prorocentrum minimum*, and the cryptophyte *Rhodomonas salina* (24, 27), almost all of which have complete thiamin biosynthesis pathways based on available genomic data for the closest relatives or experimental work. The exception is *R. salina*, which appears to lack the synthases for both precursor compounds. Quotas from these species spanned from 100 to 585 nmol g C^−1^, and free thiamin (thiamin without phosphate groups) was the dominant intracellular form (24). Methodological limitations in this prior study could result in dephosphorylation, as could stress-induced induction of intracellular phosphatases. Here, we found most intracellular thiamin was in the form thiamin pyrophosphate (Table 1).

10.1128/mBio.01459-17.6 FIG S5  Summary of currently available thiamin quotas and minimum requirements in marine phytoplankton. (A) Available thiamin quotas obtained by direct measurements that have been previously reported (black circles) or from our study (blue circles) in moles of thiamin per mole of carbon are grouped as being either from prototrophic and auxotrophic species. An environmental sample of a size-fractionated natural community is also included from the tropical eastern Atlantic. (B) Minimum thiamin requirements calculated from dose-response assays (black circles) and our direct measurement of minimum thiamin per cell (blue circles). For both panels, error bars represent SD of replicate measurements when available. Letter subscripts denote source: a, Suffridge et al. (4); b, this study; c, Paerl et al. (11); d, Tang et al. (25); e, Sylvander et al. (24); f, A. F. Carlucci and P. M. Bowes, J Phycol 8:133–137, 1972, https://doi.org/10.1111/j.1529-8817.1972.tb04013.x. Download FIG S5, PDF file, 0.1 MB. Copyright © 2017 Gutowska et al. 2017 Gutowska et al. This content is distributed under the terms of the Creative Commons Attribution 4.0 International license.

10.1128/mBio.01459-17.7 FIG S6  Cell densities of *E. huxleyi* that were used to calculate growth rates in Fig. 3B. Treatments are amended with 100 to 500 pM thiamin, HMP, or AmMP. Download FIG S6, PDF file, 0.1 MB. Copyright © 2017 Gutowska et al. 2017 Gutowska et al. This content is distributed under the terms of the Creative Commons Attribution 4.0 International license.

Cellular quotas appear to vary considerably between related taxa: for example, the diatoms *P. tricornutum* (235.7 ± 29.3 nmol g C^−1^) and *S. costatum* (423.5 ± 83.0 nmol g C^−1^) (24). This variability may reflect organismal differences in luxury storage capacity. Cell size and carbon content also are important, and when we took these into consideration, the luxury values for *E. huxleyi* (327.7 ± 110.6 nmol g C^−1^) were similar to those of several algae in the study of brackish taxa (24). It still remains unclear how these observations translate to organismal quota differences under environmentally relevant supplies where luxury storage may not be possible. Standard culture medium (luxury availability) reflects a supply level that is ~15,000 times the maximum thiamin concentration reported in the Sargasso Sea photic zone (2), while the replete level is 50 times higher. Here, despite equivalently high growth rates in the luxury and replete treatments (Table 1), *E. huxleyi* thiamin quotas under the latter (54.3 ± 4.7 nmol g C^−1^) were most similar to quotas under our limited conditions (31.6 ± 3.4 nmol g C^−1^) (Table 1). Given how different the quota is in cells grown under more environmentally relevant supply levels, our results should improve parameterization of interaction models and phytoplankton requirements.

Direct measurements of cellular thiamin quotas using the standard thiochrome assay (27, 47, 48) also allowed us to test the efficacy of the established procedure for calculating minimum cellular quotas (MCQs) (Fig. S5). MCQ calculations are based on maximum cell densities attained over a concentration gradient of thiamin/precursor amendments (11, 25). We observed a discrepancy between the MCQ values calculated from dose-response curves and those quantified using the thiochrome assay. The MCQ measurements showed that the calculated MCQs overestimate the intracellular thiamin pool. To calculate MCQ, the molar quantity of substrate in the initial medium is divided by the maximum cell density. A critical assumption is that cells exhaust the substrate supply, an assumption that appears to be violated when thiamin is provided to *E. huxleyi* (Fig. 4C; Table S1h). The measured MCQ in HMP-amended medium was also significantly lower than the calculated MCQ, but with a smaller difference than for thiamin (Fig. 4C; Table S1h). Future chemostat-based experiments with a constant supply rate (12, 26) will be important for deeper understanding of uptake kinetics and cellular quotas of thiamin-related compounds.

### Defining biochemical strategies in B_1_ community ecology.

**FIG 5:**
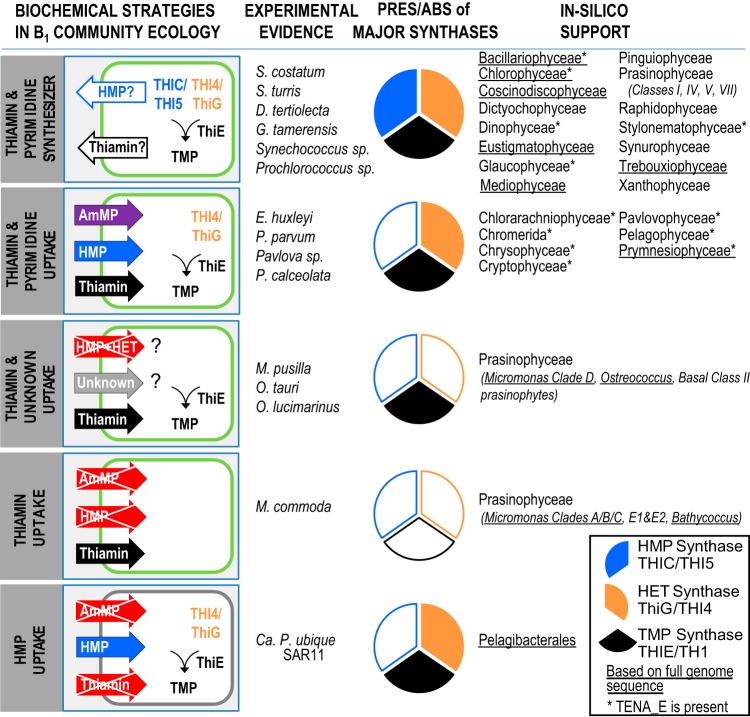
Five proposed biochemical strategies used by plankton to meet intracellular thiamin requirements. Illustrations represent eukaryotic phytoplankton (outlined in green) or bacteria (gray). Pie charts illustrate the presence or absence (PRES/ABS) of the major synthase enzymes in the thiamin biosynthesis pathway, HMP synthase (blue), HET synthase (orange), and TMP synthase (black). An asterisk on the right hand names indicates that the pyrimidine remodeling (salvage) enzyme, TENA_E, was detected. Note that proteins for transport of thiazole or thiazole-related compounds are not addressed in this figure. Thiamin and HMP can be detected in spent medium from cultures of *Skeletonema costatum* (*Bacillariophyceae*), *Stephanopyxis turris* (*Bacillariophyceae*), *Dunaliela tertiolecta* (*Chlorophyceae*), *Gonyaulax tamerensis* (*Dinophyceae*), and the cyanobacteria *Synechococcus* sp. strain WH8102 and *Prochlorococcus* sp. strain MED4 grown under replete conditions with no amendment of these compounds (2), and thus are defined as thiamin and pyrimidine synthesizers. We define other strategies as “thiamin and pyrimidine uptake” for organisms that synthesize thiamin by acquiring exogenous pyrimidine compounds (or by taking up exogenous thiamin) and possible use of presently unknown compounds as “thiamin and unknown uptake,” “HMP uptake,” and “thiamin uptake.” We placed phytoplankton groups into a strategy group when at least one member possessed the illustrated gene sets (Table S1i), although absence from transcriptomes should be treated with caution. Compounds were shown experimentally to either fulfill (arrows) or not satisfy (crossed-out arrows) exogenous requirements. The prasinophyte classes are *sensu* Duanmu, Bachy et al. 2014 (58) (Tables S1 and S2).

Complexity in thiamin-related cycling in microbial communities can be simplified by identifying taxa with specific gene sets and categorizing them in biochemically defined strategies. Phytoplankton species with complete synthesis pathways can be categorized as producers of thiamin and thiamin precursors (Fig. 5; Table S1i). For the remaining species that have “incomplete” thiamin synthesis pathways, different strategies can be defined based on compounds used to generate thiamin or meet cellular requirements (Fig. 5). Some species scavenge thiamin or specific precursors, while others have the ability to remodel compounds to generate the necessary precursors. To extend this framework to groups that have not been experimentally examined, we searched for the two precursor synthases, TMP synthase and the pyrimidine remodeling hydrolase TENA_E, in a subset of other phytoplankton in the MMETSP database. As the number of species in each of the classes represented in the MMETSP database is variable, our findings do not necessarily represent the full pathway diversity that may occur in each major lineage. Eight other phytoplankton phyla possessed TENA_E. Thus, we propose that species within the *Bacillariophyceae*, *Chlorarachniophyceae*, *Chromerida*, *Chrysophyceae*, *Cryptophyceae*, *Glaucophyceae*, *Pelagophyceae*, and *Prasinophyceae* can also exploit the strategy of pyrimidine remodeling to generate the thiamin precursor HMP. To date, pyrimidine remodeling has primarily been considered from the perspective of intracellular thiamin salvage, but diverse pyrimidine (or thiazole) analogs from the environment may be remodeled to generate thiamin precursors in phytoplankton that lack the precursor synthases. Categorization in this manner can guide study design for future investigation of species assemblages and thiamin-related interactions in marine ecosystems (49, 50).

### Summary.

Our research contributes new insights into B_1_ vitamin metabolism in marine eukaryotic algae and how it may influence overall plankton dynamics. Furthermore, differences in the enzymes present shed new light on functional biodiversity. For example, unlike thiG, which is used by many haptophytes, the THI4 enzyme used by the *Pavlovales* and *Phaeocystales* relies on iron as a cofactor, a trace metal that is often limiting in the oceans. THI4 is a single-turnover enzyme that is highly expressed in model organisms (51) because it serves as a cosubstrate. Such enzymatic differences and resulting variations in trace metal requirements highlight key nuances in how biochemical pathways shape phytoplankton strategies and influence local community structure. The conceptual framework that connects our study with prior research on B vitamins as a whole (e.g., see references 2, 6, 7, 11, 37, 41, 49, and 52) is that trafficking of essential yet limited vitamin-related resources could be an important biochemical driver that structures multispecies networks. Further exploration of this concept will require identification of the cellular transport mechanisms for vitamin and precursor molecules, additional measurements of environmentally relevant cellular quotas, and finally, the development of analytical methods for quantification and turnover rates in ocean samples. Considering the low concentrations of thiamin and HMP measured in seawater to date, our results suggest that specialization in uptake of thiamin-related precursor compounds plays important roles in the structure of ocean microbiomes and biochemical strategies of individual taxa. Our findings highlight diverse biochemical repertoires that underlie these strategies and suggest that further complexities, potentially involving links to availability of macronutrients and trace metals, await discovery.

## MATERIALS AND METHODS

### Phylogenetic analyses.

18S rRNA gene sequences were retrieved from the MMETSP (https://imicrobe.us/project/view/104) metadata files and aligned using ClustalW. Positions with gaps were masked. Maximum likelihood phylogenetic trees were computed using the general time-reversible model in MEGA6 (53). A discrete gamma distribution was used to model evolutionary rate differences among sites, and the rate variation model allowed for some sites to be evolutionarily variable. There were a total of 1,651 positions in the final data set. Data were bootstrapped using 100 replicates. Based on the topology of the 18S rRNA gene tree, each of the 28 MMETSP species was assigned to one of six established taxonomic orders. Subsequent reporting of thiamin pathway analysis uses this phylogeny to establish taxonomic grouping. A phylogenetic analysis was also performed on TENA_E and TENA_C protein sequences retrieved from the MMETSP peptide files. These were aligned using ClustalW, and gap positions were masked. Maximum likelihood phylogenetic trees were computed using the Whelan and Goldman +Freq. model in MEGA6 (53). A discrete gamma distribution was used to model evolutionary rate differences among sites. There were a total of 193 positions in the final data set. Data were bootstrapped using 100 replicates.

### Thiamin pathway analysis of haptophytes and other phytoplankton.

Fifty-eight haptophyte transcriptomes from 28 species were obtained from the MMETSP database, as were the genomes of *Emiliania huxleyi* CCMP1516 (18) and *Chrysochromulina tobin* CCMP291 (19). Peptide files predicted for all 58 MMETSP haptophyte transcriptomes were used to construct a searchable database and queried using Sequenceserver 0.8.0. Searches were performed for thiG, THI4, THIM, THIC, THID, THIE, THIL, TPK, and TENA using initial query sequences recovered from the genomes of *E. huxleyi* CCMP1516, *Chrysochromulina* sp. strain CCMP291, *Guillardia theta* CCMP2712, *Paulinella chromatophora* M0880, and *Phaeodactylum tricornutum* CCAP1055/1 (6) (Table S1a). Sequences acquired using BLASTP and TBLASTN searches were further used as queries in iterative BLASTs against the haptophyte MMETSP data set (E value of ≤10^−15^). Identity assignment for the recovered sequences was based on subsequent BLASTP and BLASTX searches against the NCBI nr database as well as the Pfam-A database with a cutoff E value of ≤10^−15^. Since lack of presence in a transcriptome-predicted proteome cannot be interpreted as proof of absence of a protein from the genome, results were grouped into a higher level of taxonomic organization to increase the robustness of the conclusions. Note that when a protein was not detected in the predicted protein set, we also searched the transcript assemblies using TBLASTN prior to categorizing the protein as “not found.” Predicted TENA proteins were further characterized by active site residues as defined in *Zea mays* and *A. thaliana* (23). A cysteine residue at the active site resulted in categorization as TENA_C. If the cysteine residue was lacking but two conserved glutamate residues were present, it was termed TENA_E.

We also performed searches for thiG, THI4, THIC, THI, THIE, and TENA in 138 predicted protein sets representing 22 marine phyla in MMETSP as well as a broader set of prasinophyte algae. This was done using a database queried using Sequenceserver 0.8.0 with query protein sequences retrieved from the genomes of *P. tricornutum* CCAP1055/1, *A. thaliana*, and *Saccharomyces cerevisiae* (Table S2).

### Determination of auxotrophy type and growth rate comparisons.

Axenic cultures of *E. huxleyi* CCMP2090, *Pavlova* sp. strain CCMP459, *Prymnesium parvum* CCMP1926, *Pelagomonas calceolata* CCMP1756, and *Tetraselmis astigmatica* CCMP880 were obtained from the National Center for Marine Algae and Microbiota (NCMA; Bigelow Laboratory, USA). Axenic *Micromonas commoda* (RCC299) (deposited at the NCMA under no. CCMP2709) and *Micromonas pusilla* (CCMP1545) were also used. For all cultures, axenicity was verified before and after experiments using the DNA stain 4′,6-diamidino-2-phenylindole (DAPI) and epifluorescence microscopy (54). Cultures were grown at 21°C on a 14-h/10-h light-dark cycle (150 µmol photon m^−2^ s^−1^ photosynthetically active radiation) using artificial seawater-based medium L1-Si (55), with a 1 × 10^−8^ M H_2_SeO_3_ amendment (final concentration) (56). Cultures were monitored using an Accuri C6 cytometer (BD Biosciences, USA). Thiamin (as thiamin HCl; Sigma-Aldrich, USA), 4-methyl-5-β-hydroxyethylthiazole (Sigma-Aldrich, United States), 4-amino-5-hydroxymethyl-2-methylpyrimidine (synthesized as described in reference 48), and 4-amino-5-aminomethyl-2-methylpyrimidine (synthesized as described in reference 57) were tested at concentrations ranging from 100 pM to 1 µM, depending on the experiment. Thiamin was not added to controls or treatments apart from the thiamin amendment; other medium components remained unaltered from the L1 medium described above.

Prior to experiments, cultures were maintained in mid-exponential growth for ≥10 generations in thiamin-replete medium (0.3 µM). At the start of each manipulation experiment, parallel cultures were grown under thiamin-replete and -depleted (no-thiamin) conditions until the thiamin-depleted culture exhibited initial thiamin growth limitation compared to the thiamin-replete culture. These thiamin-depleted cultures were then used to inoculate experiments to test auxotrophy status and experiments to determine growth rates at environmentally relevant concentrations. All experimental treatments were performed in triplicate in 50-ml tissue culture flasks containing 20 ml culture.

Thiamin and precursor auxotrophy was examined at 1 µM substrate concentrations in *E. huxleyi* CCMP2090, *Pavlova* sp. strain CCMP459, *P. parvum* CCMP1926, *P. calceolata* CCMP1756, *M. commoda* RCC299, *M. pusilla* CCMP1545, and *T. astigmatica* CCMP880. Cells from the initial thiamin-depleted culture were transferred in triplicate into fresh medium amended with thiamin, HMP, AmMP, and HET (all at 1 µM, final concentration) and a nonamended negative control with no thiamin. Cultures were transferred following each division or daily for species with division rates of >1 day^−1^ to maintain initial cell densities. Growth rates (µ) were calculated daily using the formula µ = [ln(*C*_*f*_) − ln(*C*_*i*_)]/*d*, where *C*_*f*_ represents the final cell concentration, *C*_*i*_ represents the initial cell concentration, and *d* represents the number of days.

To gain more environmentally relevant data, comparative growth rates over an amendment range of 100 to 500 pM were determined in *E. huxleyi* CCMP2090 and *Pavlova* sp. strain CCMP459. Cells from the initial thiamin-depleted culture were transferred into triplicate treatments containing thiamin, HMP, or AmMP, each at five concentrations between 100 and 500 pM, and a negative control with no thiamin or related amendment (see Fig. S6). Cell densities and growth rates were determined as stated above. Exponential growth rates were calculated from a 4-day period starting after the lag phase.

### Calculation of minimum cellular requirements.

Minimum cellular requirements were calculated for *E. huxleyi* CCMP2090 and *Pavlova* sp. strain CCMP459. Cells from the initial thiamin-depleted culture were transferred in triplicate into the treatments thiamin, HMP, and AmMP, provided at five concentrations ranging from 100 to 500 pM, as well as no amendment (i.e., no thiamin, HMP, or AmMP). Abundances were determined as described above. Cultures were grown out into the stationary phase until the growth rate was <0.05 day^−1^. The minimum cellular requirement (picomoles per cell) was calculated from the slope of the linear regression of the concentration of the amended compound plotted against maximum cell density, similar to reference 2.

### Quantification of cellular thiamin and carbon quotas.

*E. huxleyi* CCMP2090 thiamin quotas were determined in mid-exponential-phase cells grown for ≥10 generations in semicontinuous batch culture and other treatments as detailed here. The triplicate supply conditions tested were thiamin supplied at 300 nM (luxury treatment, standard f/2 medium concentration), thiamin and HMP individually supplied at 10 nM (replete conditions), as well as 500 pM thiamin and 100 pM HMP individually supplied (limiting conditions). Supply concentrations differed in limiting conditions in order to generate cultures with comparable growth rates. Cultures were transferred daily (replete) or every second day (limited) to not exceed 1 × 10^6^ cells ml^−1^. Cell densities and growth rates were determined as described above. Thiamin quotas were also quantified in triplicate cultures grown in medium amended with 500 pM thiamin or HMP and harvested at the onset of the stationary phase to replicate the condition of cells used for calculation-based estimates.

Quantification was performed using the standard thiochrome assay (33–35 [https://www.protocols.io]). Briefly, before the thiochrome assay was performed, thiamin that was external to cells was removed by centrifuging cultures (100 ml) at 4,000 × *g* for 10 min at 4°C. The supernatant was removed, the pellets were resuspended in 50 ml of thiamin-free medium, and the cycle was repeated for three rounds. After the last wash, pellets were resuspended in 1.5 ml of thiamin-free medium, and the cell number was quantified (Accuri C6 cytometer; BD Biosciences, USA). A final cell pellet was generated by centrifugation at 10,000 × *g* for 30 min at 4°C, the supernatant was removed (and cells quantified), and the pellet was cryo-frozen prior to storage at −80°C. Cell numbers in the supernatant were subtracted from those prior to final centrifugation step to determine cell counts in the pellets analyzed. Dual-parameter flow cytometric histograms of forward angle light scatter (FALS) versus chlorophyll fluorescence indicated that cellular integrity was not compromised during centrifugation (see Table S1j in the supplemental material). The deionized water used here was purified using active charcoal followed by filtration and determined to be thiamin free using the thiochrome assay (27, 47, 48). Cell pellets were suspended in 100 μl of 7% HClO_4_, sonicated for 2 min, and mixed with 50 μl 4 M CH_3_CO_2_K and 50 μl 30 mg ml^−1^ K_3_[Fe(CN)_6_] in 7 M NaOH. After 1 min, the reaction was neutralized with 6 M aqueous HCl and centrifuged, and the supernatant was analyzed by reverse-phase high-performance liquid chromatography (HPLC; Agilent 1200 series) with fluorescence detection (excitation 365 nm, emission 444 nm). A Supelcosil SPLC-18-DB column (25 cm by 10 mm, 5 μm) was used with a gradient of the following compounds: A, H_2_O; B, K_2_HPO_4_ (pH 6.6); C, CH_3_OH. The gradient structure was 0 min for 100% B, 5 min for 100% B, 14 min for 7% A–70% B–23% C, 25 min for 25% A–75% C, and 28 to 34 min for 100% B. A calibration curve in the nanomolar concentration range was constructed under the same experimental conditions by plotting the fluorescence signal peak area on the HPLC chromatogram against known concentrations of thiamin pyrophosphate (TPP), thiamin monophosphate (TMP), and free thiamin (ThF). The three forms were added to determine the thiamin pool (T sum), here referred to as the thiamin quota. *E. huxleyi* CCMP2090 cellular carbon content was measured using 20 ml of culture filtered onto precombusted GFF filters and frozen at −20°C until analysis at the Horn Point Analytical Services Laboratory (http://www.umces.edu/analytical-services).

### Statistical analysis.

Differences in growth rates and cellular quotas between thiamin and pyrimidine analog treatments were tested using one-way analysis of variance (ANOVA) and Holm-Sidak tests for *post hoc* analysis using SigmaPlot 13 (Systat Software, Inc., USA). Minimum cellular quotas were calculated from maximum cell densities in thiamin and pyrimidine analog treatments based on regression analysis.
