# Supplementary material for: Globally Important Haptophyte Algae Use Exogenous Pyrimidine Compounds More Efficiently than Thiamin
Source: mBio. 2017 Oct 10;8(5):e01459-17. doi: 10.1128/mBio.01459-17 (PMC5635689; doi:10.1128/mBio.01459-17)
Supplement: TEXT S1 [file mbo005173506s1.docx]

**Supplementary Methods**

**Phylogenetic analyses of prasinophyte and chlorophytes.** 18S rRNA gene sequences were retrieved from the MMETSP database (http://data.imicrobe.us/project/view/104) metadata files and aligned, together with additional prasinophyte and streptophyte sequences, using MAFFT. Gblocks (http://molevol.cmima.csic.es/castresana/Gblocks_server.html) was then used to eliminate any poorly aligned positions and divergent regions. There were a total of 1633 positions in the final dataset. Maximum-likelihood phylogenetic trees were computed using the General Time Reversible model in MEGA6. Data were bootstrapped using 100 replicates.

**Thiamin pathway analysis of prasinophytes and chlorophytes.** 40 prasinophyte transcriptomes were obtained from the MMETSP as were the genomes of *Micromonas pusilla* CCMP1545, *Micromonas sp* RCC299, *Micromonas sp* CCMP1764, *Bathycoccus prasinos* RCC1005, *Ostreococcus tauri* OTH95, *Ostreococcus lucimarinus* CCE9901, and *Ostreococcus sp* CCMP809. Peptide files predicted for all MMETSP prasinophyte transcriptomes were used to construct a searchable database and queried using SequenceServer 0.8.0. Searches were performed for the biosynthesis proteins THIM, THI4, TENA_E, THIE, TH1, and THIC using initial query sequences recovered from the genomes of *Coccomyxa subellipsoidea*, *Chlamydomonas reinhardii*, and *Prymnesium parvum* (Table S2(a) in the supplementary material). Sequences acquired using BLASTP and TBLASTN searches were further used as queries in iterative BLASTs against the prasinophyte MMETSP dataset (e-value ≤ 10^-5^ cut-off). Since lack of presence in a transcriptome-predicted proteome cannot be interpreted as proof of absence of a protein from the genome, results were grouped into a higher level of taxonomic organization to increase robustness of the conclusions. Pfam searches were also performed on a selection of sequences.

**Thiamin and precursor amendments in Sargasso Sea water.** *Micromonas pusilla* CCMP1545 and *Micromonas commoda* RCC299 were cultured axenically in Sargasso seawater with f/2 nutrient additions on a 13:11 light:dark cycle at 21°C and ~130 μmol photon m^-2^ s^-1^. Cells were grown exponentially for 15 transfers (~12 generations, average μ for last four transfers=0.50 ±0.10 day^-1^) under thiamin replete conditions (0.3 μmol L^-1^ thiamin). At the start of experiments, cells were centrifuged and resuspended in their respective experimental treatments. Thiamin, HMP (4-amino-5-hydroxymethyl-2-methylpyrimidine) and HET (4-methyl-5-hydroxyethylthiazole) were added at final concentrations of 1 μmol L^-1^, except for controls, where thiamin was added at 0.3 μmol L^-1^. Cell counts were determined by flow cytometry (InFlux, Becton Dickson, USA). At each time point l ml of sample was fixed with glutaraldehyde (0.25%, final concentration) and flash frozen for later analysis.

**UV degradation of thiamin.** Artificial seawater based L1-Si medium was placed directly on a table top UV (312 nm) transilluminator (Spectroline Model TVC-312R, USA) in a sterile glass container for a time course of 1 to 180 minutes. The medium contained an amendment of 500 pmol L^-1^ thiamin but did not contain the standard B_7_ and B_12_ amendments. Following UV exposure B_7_ and B_12_ were added to the medium. *E. huxleyi* cultures were grown out to maximum cell density in medium exposed to UV light over a time course of 1 to180 min to determine the optimal exposure period based on cell density. Triplicate cultures were then grown out to maximum cell density in medium exposed to UV for 30 min as well as medium with non-degraded 500 pmol L^-1^ thiamin or HMP amendments.
