# Supplementary figures and images for: Globally Important Haptophyte Algae Use Exogenous Pyrimidine Compounds More Efficiently than Thiamin
Source: mBio. 2017 Oct 10;8(5):e01459-17. doi: 10.1128/mBio.01459-17 (PMC5635689; doi:10.1128/mBio.01459-17)

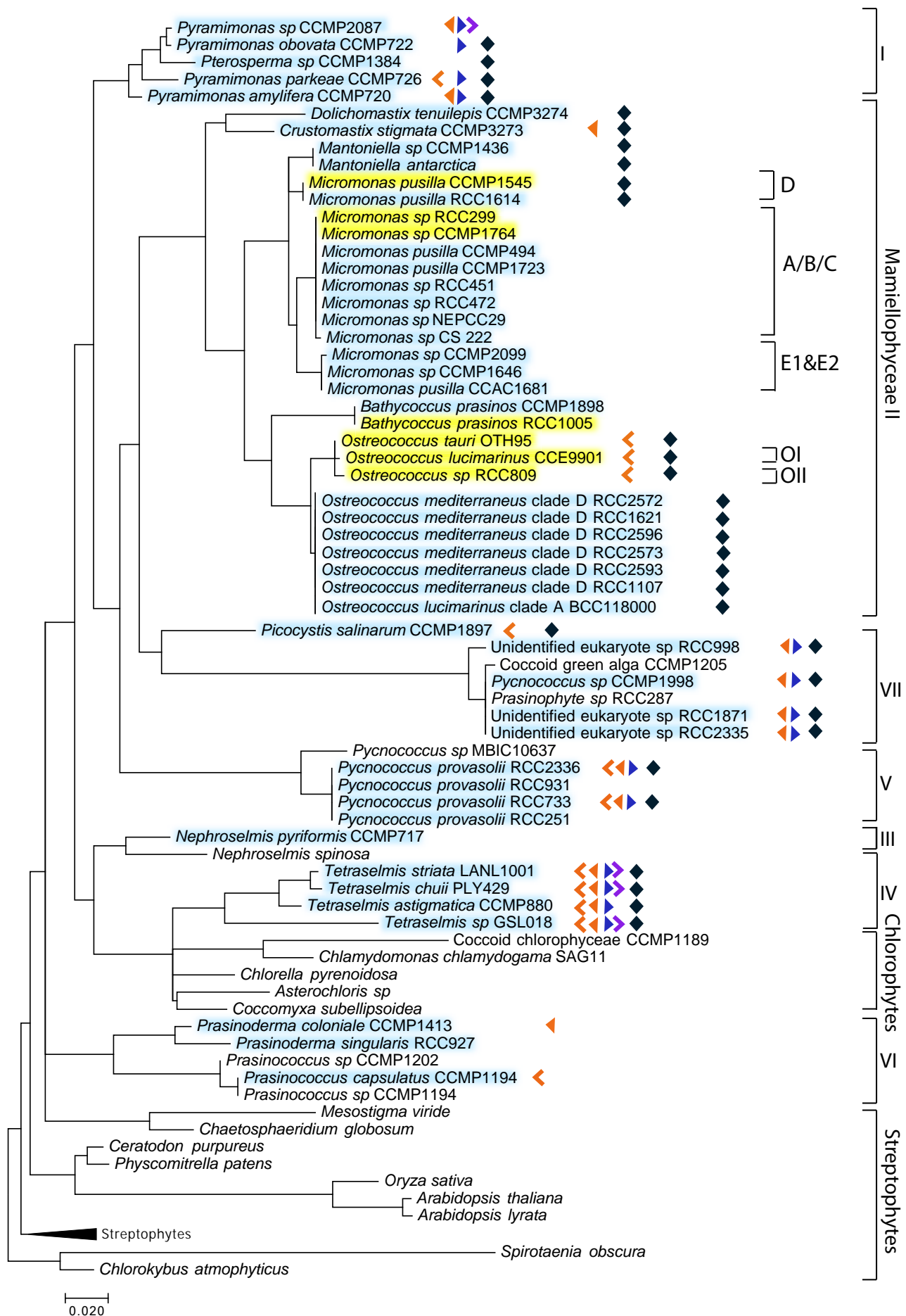

◀ THIM      ▶ TENA\_E      ◆ THIE/TH1  
 ◀ TH14      ▶ THIC

Supplement: FIG S1 [file mbo005173506sf1.pdf]

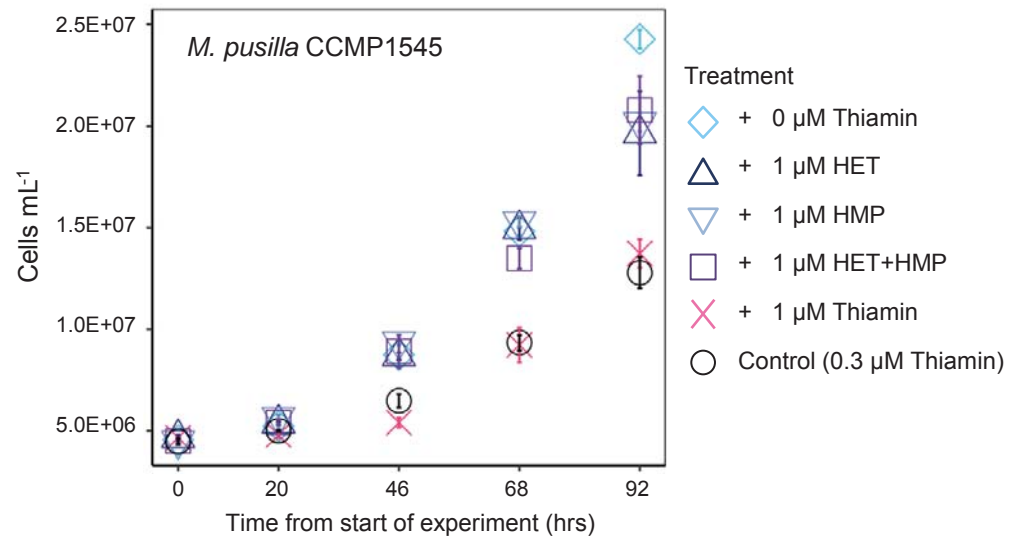

Supplement: FIG S2 [file mbo005173506sf2.pdf]

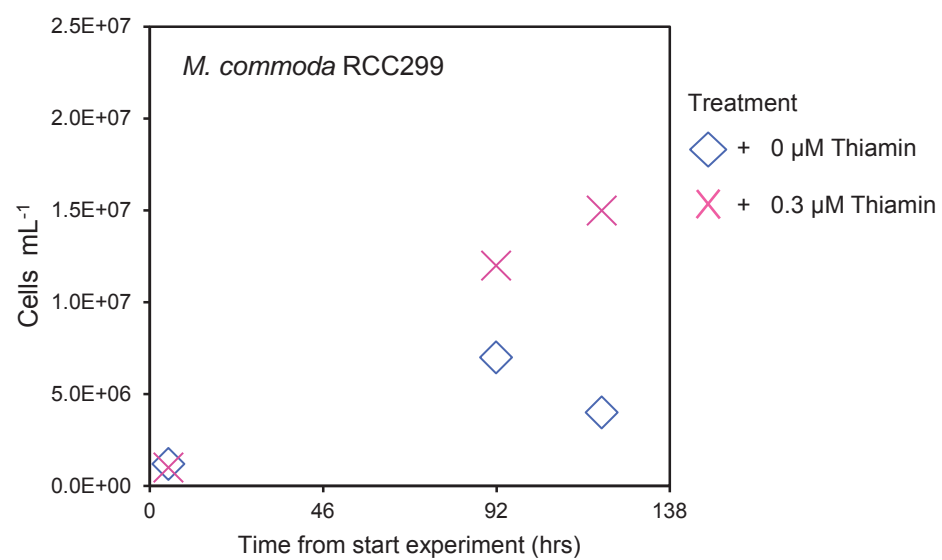

Supplement: FIG S3 [file mbo005173506sf3.pdf]

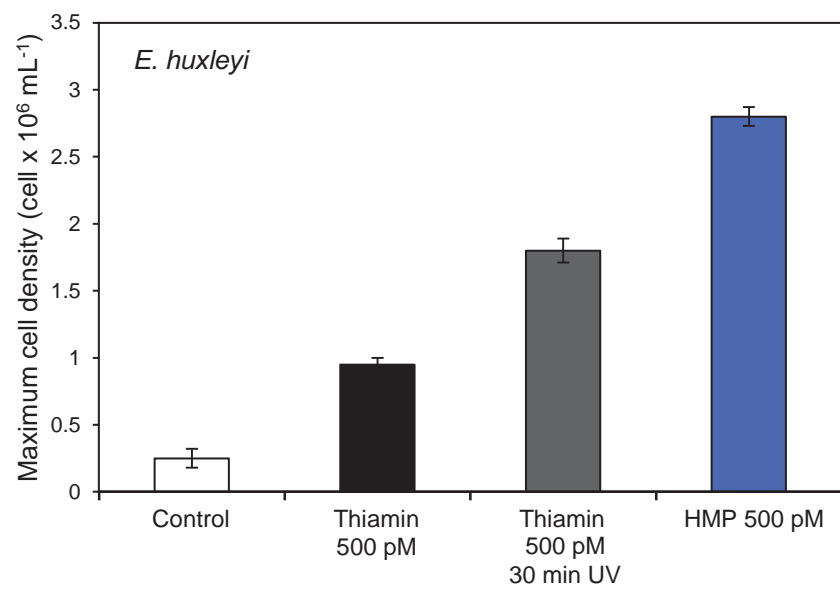

Supplement: FIG S4 [file mbo005173506sf4.pdf]

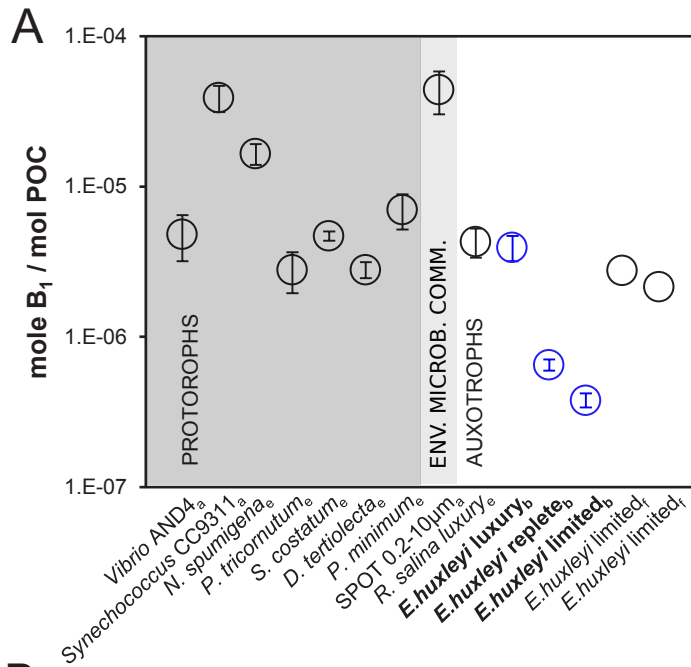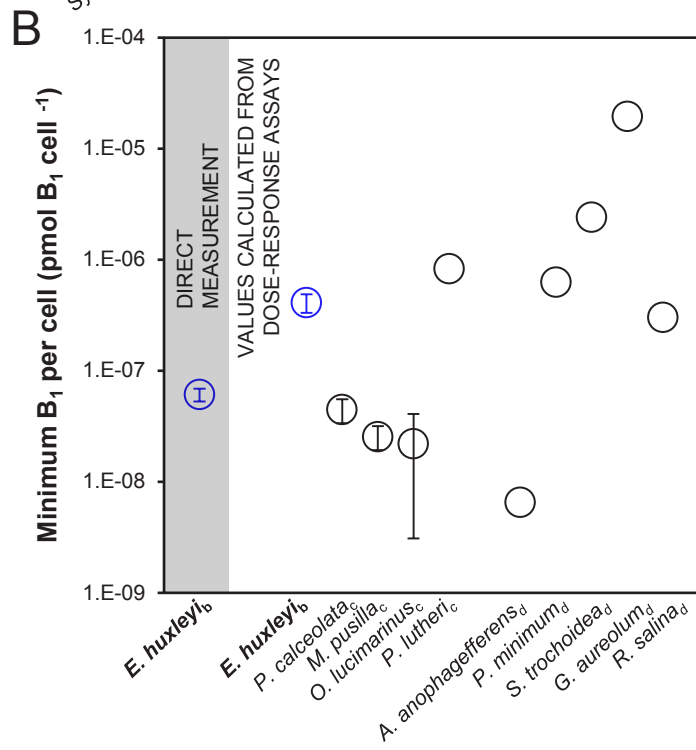

Supplement: FIG S5 [file mbo005173506sf5.pdf]

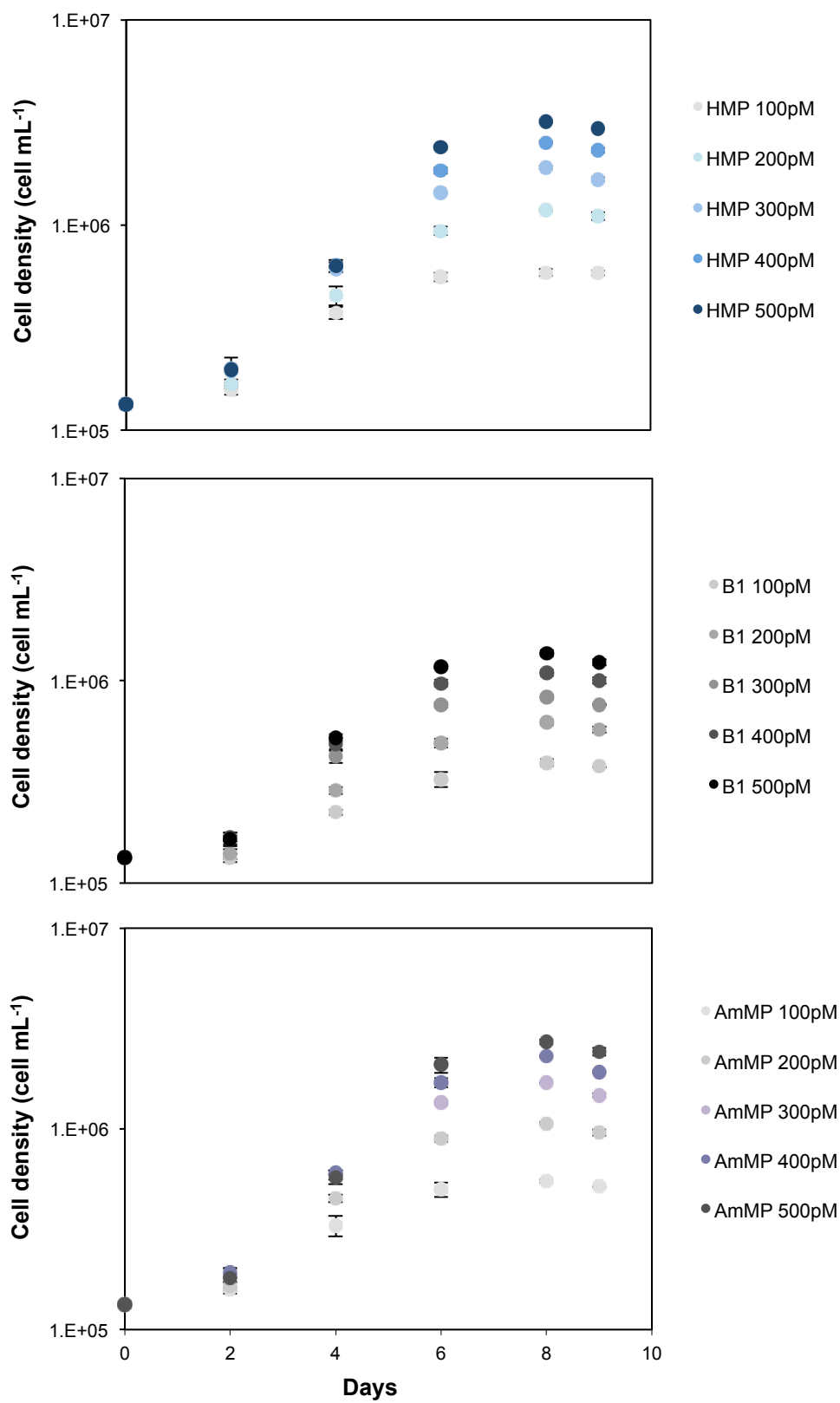

Supplement: FIG S6 [file mbo005173506sf6.pdf]
